# Risk factors and association of body composition components for lumbar disc herniation in Northwest, Mexico

**DOI:** 10.1038/s41598-020-75540-5

**Published:** 2020-10-28

**Authors:** Adriana G. Mateos-Valenzuela, Mirvana E. González-Macías, Silvia Ahumada-Valdez, Carlos Villa-Angulo, Rafael Villa-Angulo

**Affiliations:** 1 Laboratory of Bioinformatics and Biofotonics, Engineering Institute, Autonomous University of Baja California, Mexicali, Baja California México; 2 Faculty of Sports, Autonomous University of Baja California, Mexicali, Baja California México

**Keywords:** Biotechnology, Health care, Risk factors

## Abstract

The goal of this study was to investigate the association of body composition components and to elucidate whether any of these components is a risk factor for Lumbar Disc Herniation (LDH). The group of study consisted of 90 adults involved in a physical activity program due to overweight and obesity. 19 adults with medical diagnostic through Magnetic Resonance Imaging with LDH. Body composition data was obtained with a bioelectrical impedance analyzer. Descriptive statistics and principal components analysis permitted to analyze the information's structure and to visualize information clusters. A logistic regression analysis allowed us to find the association between some of the variables of body composition with LDH. The Degree of Obesity, Body Mass Index, Visceral Fat Area and the Abdominal Circumference resulted associated (*P* values of 0.0388, 0.0171, 0.0055 and 0.0032, respectively). The application of Odd Ratio allowed us to declare the Visceral Fat Area and Abdominal Circumference as risk factors to develop Lumbar Disk Herniation. Our results provide a new record for future studies, and support for prescription of physical activity and changes in diet, to correct or prevent the development of LDH in the population of Baja California.

## Introduction

Obesity and overweight are worldwide public health subject. They have been associated as risk factors for many current aliments^1^. Specially, the Lumbar Disc Herniation (LDH) is a musculoskeletal condition that has been associates to obesity and overweight, along with genetic, environmental and those related with lifestyle condition^2^. Nowadays, LDH has a prevalence over 23.12% in Mexico^3^. And, it is the second cause of work incapacity in the United States of America^4^.

Results from a study carried out by Han et al.^5^, showed that the prevalence of the lumbar disc herniation and lower back pain increase when, both men and women, population present an increase in abdominal circumference (AC) of the relation waist-hip and the Body Mass Index (BMI). The BMI and AC have been utilized as methods to evaluate overweight and obesity. Actually the increased values of these measurements are referred to as predisposal factors to LDH. It also has been suggested that metabolic factors associated with obesity can determine an LDH^6^. A study made in Finland by J. Takatalo et al.^7^, regarding the association of abdominal obesity and the deterioration of the lumbar disc, using Magnetic Resonance Imaging (MRI) and bioelectrical impedance analysis (BIA) as methods to measure the body composition, concluded that the measurement of abdominal obesity and the waist circumference are associated with disc degeneration in adults.

Mexico is a country with high rates of overweight and obesity^8^. Especially, in the state of Baja California, 31.9% of adult population (older than 20 years old) are overweight, while the 39.5% are obese (data obtained from the National Poll of Health and Nutrition ENSANUT in 2012). A study carried out by Zonana-Nacach in 2013^9^, in the registry of the Social Security of municipalities from Baja California, showed a LDH incidence of 46% in people with a mean age of 41.8 years old, women being the most affected, primarily with LDH in the lumbar 4 and 5.

In this study, we aimed to investigate the association of body composition components for Lumbar Disc Herniation (LDH), and to elucidate whether any of the components is a risk factor for developing LDH. Study population consisted of persons involved, by medical prescription, in a physical activity program at the Aquatic Complex of the Autonomous University of Baja California, in Mexicali, Baja California, referred to by medical prescription. Besides medical history and questionnaires of lifestyle conditions applied to patients diagnosed with LDH, the information consisted of Body composition measures taken with the Electrical Bio-impedance method. Descriptive statistics and principal components analysis permitted to elucidate the information's structure and to visualize information clusters. A logistic regression analysis revealed the association between some of the variables of body composition and LDH. Finally, the application of Odd Ratio allowed declaring some of the variables as risk factor for the development of LDH in the population of Mexicali, Baja California.

## Results

**Figure 1:**
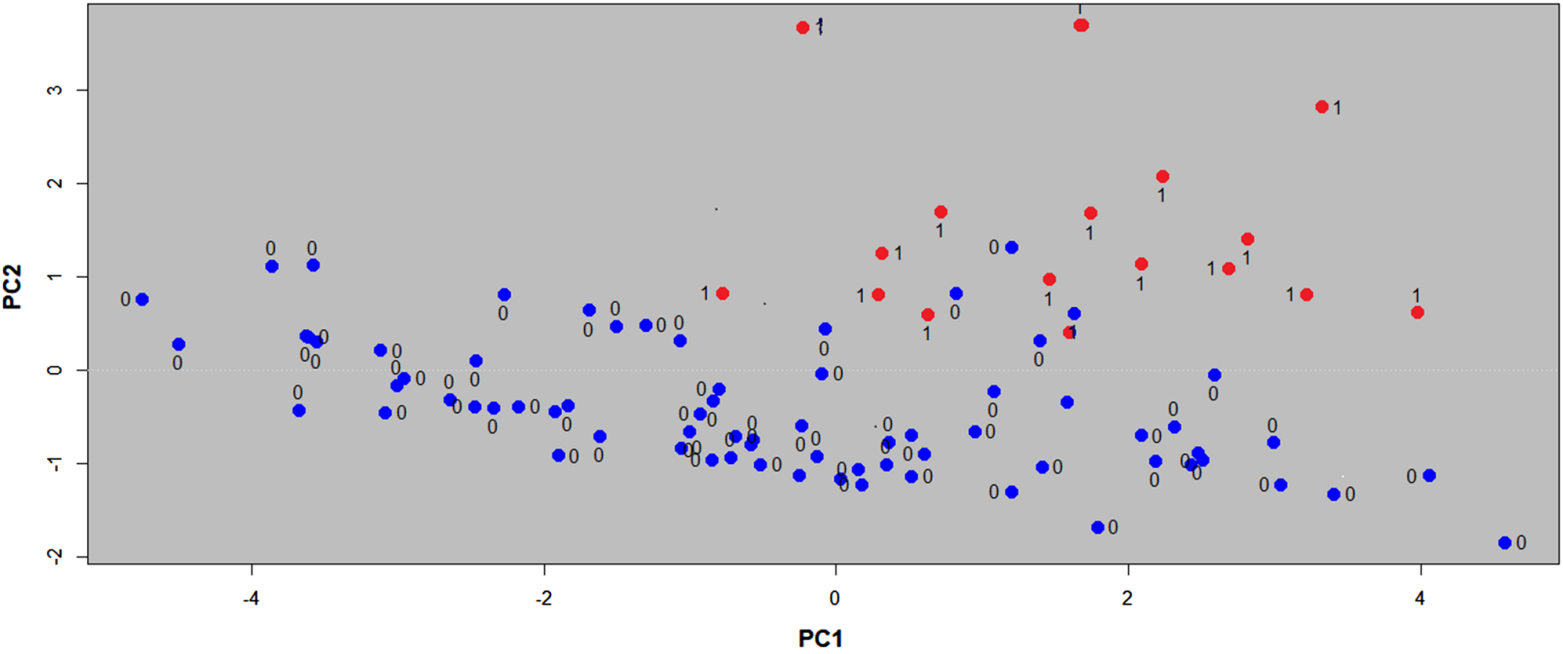
Principal components analysis (PC1 vs PC2). Red dots (labeled 1) corresponds to individuals with LDH. Blue dots (labeled with 0) corresponds to users without LDH. There is a clear differentiation between both groups of individuals. Using R software (https://www.R-project.org/).

The software R version 3.2.3^10^ was used to carry out the analysis of the data. First, the 90 vectors generated for individuals (see Data recollection in the Method section) were shifted to be centered at zero and then normalized to obtain a unitary variance. To analyze the data structure and look for differentiation, a Principal Components Analysis was applied to the resulting vectors. Figure 1 shows the plot of Principal Components 1 (PC1) against Principal Component 2 (PC2). Red dots (labeled with 1) correspond to individuals with LDH, while blue dots (labeled with 0) correspond to individuals without LDH. As can be seen on Fig. 1 the plot shows a clear differentiation between both groups. Individuals with LDH are concentrated in the right superior part of the graphic; with loads from −1 to 4 for PC1, and from 0 to 4 for PC2. While individuals without HDL appear dispersed from -5 to 5 for PC1, and from -2 to 1.5 for PC2.

### Association analysis and risk factor definition

In order to investigate if there is association between LDH and any of the body composition components, we implemented Logistic Regression Analysis. We defined a dependent variable *Y*_*i*_ for each individual *i*; assigning 1 if the individual had LDH, and 0 if the individual did not have LDH. Next, we defined six explicative variables *x*_*i*_ for each individual *i*. Each *x*_*i*_ was assigned with the value of Degree of Obesity (DO), Visceral Fat Area (VFA), Body Mass Index (BMI), Abdominal Circumference (AC), Body Lean Mass (BLM), and Body Fat Mass (BFM) measure, respectively. After applying the Logistic Regression, statistical significance (*P* values) was computed to measure the level of association of each explicative variable with the dependent variable. The Degree of Obesity and the Body Mass Index resulted associated with a *P value* of 0.03878 and 0.01707, respectively. The Visceral Fat Area and the Abdominal Circumference resulted highly associated with a *P value* of 0.00554 and 0.00316, respectively. And, Body Lean Mass and Body Fat Mass resulted no associated.

To define risk factors, all variables that resulted significant in the association analysis (OD, VFA, BMI, and AC) were inspected. The Odds Ratio (OR) computation and Risk Factor definition criteria, as described by Szumila et al. 2010^11^ was applied. From results, statistical significance, OR value, and OR confidence interval of 95%, for each variable were inspected. Then, all variables that satisfied the next criteria were declared a risk factor: (1) if the variable resulted statistically significant (*p value* ≤ 0.05); (2) Odd Ratio was different than 1; and (3) Odd Ratio Confidence Interval (95% CI) did not include 1. Then, if a variable satisfied these three conditions, and it's OR > 1, therefore it was declared as a risk factor associated with higher odds of LDH. In the same way, If the variable satisfied the three conditions, and its OR < 1, therefore it was declared as a risk factor associated with lower odds of LDH. Table 1 shows the results. First column presents the variable (body composition component). Second column presents the Odd ratio and its 95% CI. Third column presents *p* value, and the fourth column presents the declaration of Risk Factor.

**Table 1:** LDH risk factors definition.

Variable	Odds ratio (95% CI)	*p* value	Risk factor
Degree of obesity (DO)	1.226e−06 (7.393e−01 to 1.619)	0.03878	No
Visceral fat area (VFA)	8.974e−01 (8.171e−01 to 0.968)	0.00554	Yes, associated with lower odds of LDH
Body mass index (BMI)	2.636e−01 (6.811e−02 to 2.899)	0.01707	No
Abdominal circumference (AC)	1.415e+00 (1.159e+00 to 1.807)	0.00316	Yes, associated with higher odds of LDH

A variable was declared as Risk Factor if its *p* value ≤ 0.05, its OR was different than 1, and its OR 95% CI did not include 1.

As we can see in Table 1, VFA resulted as a risk factor, associated with lower odds of LDH. While AC resulted as a risk factor associated with higher values of LDH.

## Discussion

Results obtained in this study show association of Degree of Obesity, Visceral Fat Area, Body Mass Index and Abdominal Circumference with the Lumbar Disc Herniation in adults from Baja California, Mexico. Most of the individuals in the study were in a condition of overweigh and obesity (85.22%), implying possible alteration of lumbar disc properties, because the increased mechanical load, as mentioned by Iatridis et al.^12^. In support of our results, overweight has been associated to lumbar disc degeneration at histological and macroscopic level, causing changes in the structure of the disc and affecting the regeneration process^13,14^. Takatalo et al.^7^, measured body adiposity using Magnetic Resonance Imaging, and an Inbody corporal composition analyzer. Their results showed association between degenerated disk and abdominal obesity; which is consistent with our results, where 79.44% of individuals presented high values of Visceral Fat Area. In similar studies performed in Israel, Hershkovich et al.^15^ found association between overweight and obesity with low back pain, which has been associated with disk degeneration.

After Odd Ratio criteria, Visceral Fat Area and Abdominal Circumference were declared as Risk Factors for suffering from Lumbar Disc Herniation in individuals from our study population. Previous studies have mentioned the prevalence of low back pain (characteristic of herniated lumbar disc coupled with an irradiated pain in one of the lower limbs) as risk factors in patients with high BMI. In addition, the increase in adipose tissue has been highly associated with the body´s biomechanics and damages in the spine^16^. From structural damages, we can find those of Modic change (pathological changes in the vertebral body)^17^. Risk of damage is mainly present in people with android obesity, while a protection factor for sever damage is present in people with gynoid obesity^18,19^. Besides the distribution of the body adiposity can play a more important role in triggering lumbar disc herniation^20^.

Others researches such as Samartizis et al.^21^ found that overweight and obesity are Risk Factors for the presence, extension, and overall severity of disc degeneration. It is worth mentioning that the values of BMI related to disc degeneration, has many limitations, so an analysis of body composition is more accurate, as it says about the distribution of fat, particularly abdominal fat^22^. Takatalo et al.^7^, performing body adiposity measurements using magnetic resonance imaging, found very similar abdominal circumference measurements, so they proposed the clinical use of this measure to evaluate the abdominal adiposity as a Risk Factor for disc degeneration, with the advantage of being a method of easy access and low cost. Other researches like Han et al.^5^ showed that the prevalence of chronic low back pain and lumbar disc herniation are increased when individuals of a population present an increased in abdominal circumference, and a high BMI. However there is a higher relative risk of lumbar pain in those people with increased abdominal circumference than those with high BMI.

This study aimed to identify body composition components as risk factors for LDH. Its main limitation resides in the sample size, which was imposed by the restriction of individuals involved in the physical activity program at the aquatic complex of the UABC.

## Conclusion

The four body composition components (Degree of Obesity, Visceral Fat Area, Body Mass Index, and Abdominal Circumference) that resulted associated with the Lumbar Disc Herniation are strongly related to body adiposity distribution. Visceral Fat Area and Abdominal Circumference were declared as risk factors for developing Lumbar Disk Herniation in a population sample of Baja California, adding up to previous existing risk factors, such as the age of the person, mechanical, and genetic factors.

An advantage of using Visceral Fat Area and Abdominal Circumference as risk factors is that they can be prevented, or corrected under traditional treatment or exercising program, while age, mechanical and genetic factors are not correctable. Finally, our results provide a new record for future studies, and support for prescription of physical activity and changes in diet, looking for decreasing the body adiposity and increasing muscular area of the core, in order to correct or prevent the development of Lumbar Disc Herniation in the population of Baja California.

Besides, this is the first study reporting body components as risk factors for Northwest Mexican population. Prevalence of LDH (21.11%) resulted similar to a previous study for all Mexico (23.12%), in which just prevalence was analyzed. Further studies may focus on a bigger population in order to increase certainty.

## Materials and methods

### Ethical issues and description of population

The participants were informed about the objectives and were included only after reading and signing the written informed consent statement. Consent and the research protocol was approved by the Engineering Institute of the Autonomous University of Baja California (UABC) research ethics committee, following the principles regarding human experimentation proposed by the Helisinki declaration.

The group of study consisted of 90 adult from which 78 are women and 12 are men with a mean age of 57.4 ±11.51 years old. All people included were patients of a physical activity programs at the Aquatic Complex of the UABC in Mexicali, Baja California. Exclusion criteria include individual with an age less than 18 years old. In the group of study, 19 adults with medical diagnostic through MRI with LDH. Given that targeted people consisted only in patients referred to the physical activity program by medical prescription, the population size was limited. Therefore, the sampling technique was non-probabilistic sampling by convenience of number of patients available.

### Data recollection

A three-step procedure was used to collect information: first, we requested the medical history, personal data, family background, pathological and non-pathological background (condition and current treatment). These information is in addition to the questionnaires Oswestry^23^. Second, blood glucose (glucometer, SD check) and pressure (stethoscope and manual Baumanometer, Welch Allyn) was taken. The height of each individual was measured using a Stadiometer graduated in centimeters, with a scale from 0 to 250 (SECA) [Medical Scales Measuring System]. Finally, using the InBody 720 equipment (Biospace Co., Ltd) the body composition was captured.

With the captured information, a 95 variables vector was generated for each individual; of which 41 variables were from Clinical History and 54 variables from body analysis. The variables of our interest were: the presence of herniated disc in the lumbar region and those obtained by the Body Analyzer (InBody 720), which are: the Degree of Obesity (DO), Visceral Fat Area (VFA), Body Mass Index (BMI), Abdominal Circumference (AC), Body Lean Mass (BLM) and body fat mass (BFM). Table 2 shows the descriptive statistics for the variables of our interest.

**Table 2:** Analysis of descriptive statistics, of DO, VFA, BMI, AC, BLM and BFM.

Variable	Mean (95% CI)	SD	MAX	MIN
Degree of obesity (%)	143.62 (143.44–143.82)	28.52	221.69	92.65
Visceral fat area (cm^2^)	131.87 (131.64–132.10)	34.56	213.12	38.81
IMC (kg/cm^2^)	31.05 (31.01–31.09)	5.99	47.69	19.91
Abdominal circumference (cm)	104.42 (104.34, 104.52)	13.68	140.2	69.8
Body lean mass (kg)	24.56 (24.53–24.59)	4.45	38.73	17.13
Body fat mass (kg)	34.02 (33.95–34.10)	11.11	57.80	12.6

### Principal component analysis

The central idea of PCA is to reduce the dimensionality of a data set, which consists of a large number of interrelated variables, while retaining as much as possible of the variation present in a data set. This is achieved by transforming a new set of variables, the main components (PC’s), which are not correlated, and are arranged in such a way that the former retain the greatest variation present in all the original variables.

Formally PCA is defined as an orthogonal linear transformation, which transforms the data to a new coordinate system such that the highest variance for any data projection lies in the first coordinate (called the first principal component), the second largest variance in the second coordinate and so on. PCA is theoretically the optimal transformation for a given data set, in terms of least squares. The procedure to obtain the main components can be summarized as follows: Given an X^T^ vector of n dimensions, X = [x_1_, x_2_ …, x_n_]^T^, of which its means vectors, M, and covariance, C, are described by: M=E(X)= [*m*_*1*_, *m*_*2*_, *…*, *m*_*n*_]^T^ and C = E [(X − M) (X − M)^T^]. Calculate eigenvalues λ_1_, λ_2_, …, λ_n,_ and eigenvectors *P*_*1*_, *P*_*2*_, …, *P*_*n*_; and sort them according to their magnitude λ _1_ ≥ λ_2_ ≥ ⋯ ≥ λ_*n.*_ Select *d* eigenvectors to represent las *n* variables, *d* < n. Then *P*_*1*_, *P*_*2*_, *…*, *P*_*d*_ are named principal components^24^.

### Logistic regression analysis

In general, Regression Models are mathematical techniques for modeling the quantitative stochastic relation between a variable of interest and one (or a set) of explanatory variables. Specifically, these models can be expressed as follows:

$$ Y_{i} = \beta_{0} + \beta_{1} X_{1i} + \beta_{2} X_{2i} + \cdots + \beta_{p} X_{pi} + \varepsilon_{i} $$

where *Y*_*i*_: dependent variable, *X*_*1i*_, *X*_*2i*_, …, *X*_*pi*_: explicative variables or regressors, *β*_*0*_: Intersection or constant term, *β*_*1*_, *β*_*2*_, …, *β*_*p*_: Parameters, measure the influence that the explicative variables have, up on the Dependent Variable. *p*: Number of independent parameters to bear in mind. *ε*: Error of the observation due to non-controlled variables. *i*: *1*, *2*, *…*, *n:* Number of observations of variables.

With this models is possible to study the linear relationship between multiple variables and the effect that these have up on the dependent variable. *β*_*i*_ are estimated following least square criteria:

$$ \mathop {\min }\limits_{\begin{subarray}{l} \beta \in n \\ i = 1, \ldots ,n \end{subarray} } \sum\limits_{i = 1}^{n} {\left( {Y_{i} - \beta_{0} - \beta_{1} X_{1i} - \beta_{2} X_{2i} - \cdots - \beta_{p} X_{pi} } \right)}^{2} $$

And least square estimators are obtained from:

$$ \beta = \left( {X^{T} X} \right)^{ - 1} X^{ - T} Y $$

Particularly, logistic regression is a type of regression analysis used to predict the outcome of a categorical variable (a variable that can adopt a limited number of categories) based on independent or predictor variables. It is useful to model the probability of an event occurring as a function of other factors. Logistic regression analysis is framed in the set of Generalized Linear Models (GLM) that uses the function logit as a link. The probabilities that describe the possible outcome of a single trial are modeled, such as a function of explanatory variables, using a logistic function as follows^25^:

$$ \log it(p_{i} ) = \ln \left( {\frac{{p_{i} }}{{1 - p_{i} }}} \right) = \beta_{0} + \beta_{1} X_{1i} + \beta_{2} X_{2i} + \cdots + \beta_{p} X_{pi} + \varepsilon_{i} $$

where

$$ p_{i} = E\left( {\frac{{Y_{i} }}{{n_{i} }}\left| {X_{i} } \right.} \right) $$
